# Effects of a Psychological Preparation Intervention on Anxiety Associated with Pediatric Anorectal Manometry

**DOI:** 10.1155/2019/7569194

**Published:** 2019-01-01

**Authors:** Katherine Lamparyk, Lori Mahajan, Christopher Lamparyk, Ashley Debeljak, Laura Aylward, Kimberly Flynt, Rita Steffen

**Affiliations:** ^1^Cleveland Clinic Children's Hospital, USA; ^2^UH Cleveland Medical Center, USA; ^3^Wright State School of Professional Psychology, USA; ^4^Rosalind Franklin University of Medicine and Science, USA

## Abstract

**Background and Aims:**

High-resolution anorectal manometry (HRM) is associated with significant patient and parent anxiety, which can impact the success and efficiency of the procedure. The nature of HRM necessitates cooperation of an alert child. This study examined effects of psychoeducation intervention on decreasing procedural distress in both pediatric patients and their parents.

**Methods:**

A prospective randomized study of children aged 3–12 years, undergoing HRM, was performed utilizing child-centric educational video. Patients received either psychological preparation intervention or treatment-as-usual. Distress was assessed through self-reported and parent-reported anxiety measures (STAIC-S; STAI-S), physiological arousal measurements, and an observational scale of procedural distress (PBCL).

**Results:**

A total of 63 children, aged 3–12 yrs (6.7 ± 2.5), completed the study. Measures of observed and reported distress and anxiety (PBCL; STAIC-S) were significantly less in children receiving intervention. Parents of children in the intervention group also reported significantly less preprocedural anxiety (STAI-S). Effects on physiological arousal were mixed, with significant preprocedural decrease in systolic blood pressure but no difference in heart rate from baseline.

**Conclusions:**

Preprocedural psychological preparation was effective in decreasing pediatric patient and parental self-reported anxiety associated with HRM. Intervention decreased physician time necessary to successfully complete the study and significantly decreased the number of times patients had to endure balloon inflation.

## 1. Introduction

Pediatric procedural anxiety and behavioral distress is a common occurrence across medical procedures and can have longstanding negative effects [1–3]. Few studies have examined procedural distress pediatric patients undergoing gastroenterology procedures. A thorough review of the literature revealed limited studies performed to date in children undergoing endoscopic procedures [4–8]. Psychological preparation was shown to have beneficial effects. While anorectal manometry has recently been shown to result in significant preprocedural distress in children [9, 10], no studies have yet been performed to evaluate nonmedical interventions to mitigate associated anxiety.

High-resolution anorectal manometry (HRM) is a valuable tool to assess for the presence of the recto-anal inhibitory reflex (RAIR) and to assist in evaluation and optimization of therapy in children with chronic constipation [11, 12]. While the RAIR can be assessed even under general anesthesia, evaluation of sensory volumes and a patient's ability to coordinate abdominal and pelvic floor musculature is ideally completed in a conscious and cooperative state [12]. Therefore, it is especially important to reduce the procedural distress for this population.

The primary aim of this study was to determine if a psychological preparation intervention would decrease perceived and measured procedural distress in patients undergoing pediatric anorectal manometry. The secondary aim of the study was to evaluate the impact of the procedure on parental anxiety and overall satisfaction.

## 2. Methods

### 2.1. Participants

Patients undergoing outpatient high-resolution anorectal manometry for evaluation of chronic constipation at Cleveland Clinic Children's Department of Pediatric Gastroenterology were prospectively enrolled in the study. Patients aged 3 years through 12 years were included. Subjects were excluded from the study if they had previously undergone anorectal manometry or prior anorectal surgery, had a diagnosis of developmental delay, were on antianxiety medication, or were scheduled under anesthesia by the ordering staff gastroenterologist.

### 2.2. Study Design and Procedure

The following study protocol was approved by the Pediatric Institute Review Committee and subsequently the Institutional Review Board of the Cleveland Clinic. Prior to arriving for the procedure, all patient caregivers received routine instruction from the ordering gastroenterologist and written bowel preparation instructions. All patients also received a routine previsit nursing phone call to review the procedure, answer any questions, and discuss the clean-out preparation required. Parents were informed they would be permitted in the procedure room if they wished to remain with their child and encouraged to bring in a comfort item or toy from home for their child to utilize during the study.

After patients arrived and checked in for the procedure, written, informed consent was obtained from the parent or legal guardian of the patient, and verbal assent was obtained from the child per Cleveland Clinic protocol. Patients were then randomized into one of two groups.

Following randomization, the control group received treatment-as-usual, which included standard instructions and explanation of the study from the nurse and pediatric gastroenterologist. Parents and patients in the intervention group additionally viewed a psychological preparation video, specifically designed and created for this study. The video was approximately six minutes in duration. The psychoeducational and procedural content of the video was developed by a doctoral-level psychologist and pediatric gastroenterologist specializing in high-resolution manometry. A graphic designer with medical training created the video using still-life photography provided by the Cleveland Clinic Art Department, with animation and voice-over narration utilizing. The video was specifically designed using standard early childhood educational techniques to engage young learners in a playful manner. Child-like intonation, simple vocabulary, bright colors, playful fonts, and pop-ups with a reinforcing and calming message were used throughout.

The initial 3.5 minutes of video content provided age appropriate education about what to expect during the procedure as detailed from a child's perspective. A four-year-old female actress and parents were followed from check-in to procedure completion. At the beginning of the video, the procedure itself was defined. Larger vocabulary that they would encounter was phonetically broken down (e.g., manometry into “ma-nom-uh-tree”). Basic anatomy of the anorectum and the goals of the procedure were explained in child-like language. The child was educated about what would occur during the procedure and what sensations to expect. The video included views of the exact room and equipment that would be utilized, medical personnel they would encounter, and appropriate modeling of a child actor undergoing the procedure while her parents provided desirable support behaviors. The child was intentionally pictured modeling a comfortable, cooperative, and relaxed demeanor.

The final 2.5 minutes of the video focused on psychological preparation for the caregivers related to their role in the procedure. The video provided suggestions for the caregivers to best support their child during the procedure along with parental modeling from the actors. Specific instructions were given to increase desired behaviors (e.g., engaging child in distraction, remaining calm themselves) and discourage undesired behaviors (e.g., drawing attention to the procedure, overt comforting). Rationale for these recommendations was provided throughout the video.

### 2.3. Measures

Parents of both groups were administered the State-Trait Anxiety Inventory-State (STAI-S) [13] immediately prior to their child undergoing the manometry procedure, and children aged eight and older completed the State-Trait Anxiety Inventory for Children-State (STAIC-S) [14] at that time. The STAI-S and STAIC-S are validated, standardized questionnaires measuring momentary anxiety in adults and children, respectively [13, 14].

Physiological arousal was assessed by measurement of heart rate and systolic blood pressure. These measurements were compared with baseline measurements taken at the child's prior medical appointment, obtained via record review for those subjects of whom this information was available. The differences between baseline measurement and preprocedural measures were used as measures of physiological arousal.

Following completion of vital sign and anxiety measurements, both intervention and control group patients underwent high-resolution anorectal manometry (ManoScan) with sensation testing as tolerated, per standard protocol. The procedure was conducted by the same staff pediatric gastroenterologist and assisted by the same nurse for all subjects.

Upon completion of the procedure, the gastroenterologist completed the Procedure Behavior Check List (PBCL) [15]. The PBCL is a validated observational rating scale, assessing child distress behaviors. The checklist consists of 8 behaviorally anchored items, ranked on a Likert scale, with higher scores representing increased distress.

Immediately following the procedure, the child's caregiver completed a questionnaire on demographic data, caregiver observed measure of distress during the procedure, and overall satisfaction with procedure. Observed distress and overall satisfaction were measured on a seven-point Likert-type scale with higher scored indicated increased observed distress and satisfaction, respectively.

### 2.4. Analysis

Demographic variables are described with descriptive statistics, including mean and standard deviation for continuous variables and percentages for categorical variables. Differences between groups for all demographic variables are calculated with a chi-square statistic for categorical variable and independent samples t-test for continuous variables. To analyze the primary and secondary aims of the study, independent sample t-tests are conducted to compare the two groups and Cohen's d statistic was calculated to estimate the effect size.

## 3. Results

**Figure 1:**
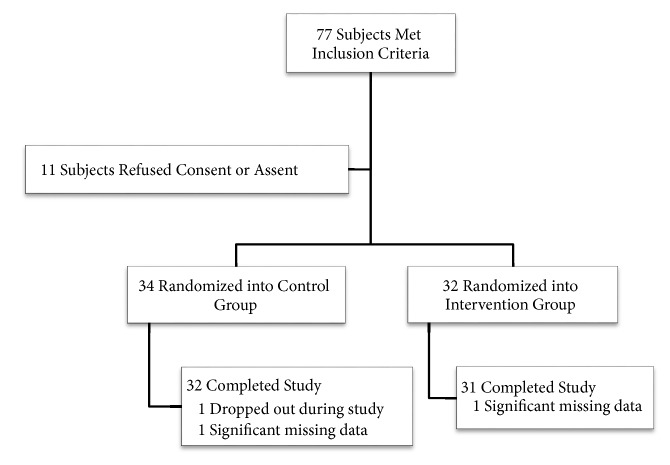


Subjects were recruited into the study over a period of 20 months. Of the 79 subjects who met inclusion criteria, 79.7% were enrolled and successfully completed the study (Figure 1). Of those excluded prior to randomization, two were found to have significant developmental delay and eleven were excluded due to failure to render either caregiver consent and/or patient assent. After randomization, one subject from the control group dropped out prior to completion of the study. Later, two subjects, one from each group, were excluded due to significant missing data. One subject from the intervention group was unable to successfully complete the manometry procedure due to behavioral problems and this child's data was included in that analysis for those items completed (e.g., preprocedural anxiety measures).

Of the 63 children who completed the study, 57% were male. Subjects ranged in age from three through twelve (*M*_*age*_ = 6.68 years, +- 2.49 years), with a majority of the sample self-identified as Caucasian (73%). A vast majority of the subjects were accompanied by their mother (95%), while 41% were accompanied by more than one caregiver. Psychiatric comorbidity was present in 14% of the sample, with all patients presenting with either a diagnosis of Attention-Deficit Hyperactivity Disorder (N = 6) or Anxiety (N = 5). There were no significant differences in demographics between the two groups (Table 1).

Children aged 8 years and older who were able to report on their own anxiety levels indicated significantly less anxiety in the intervention group than the control group (STAIC-S, p = 0.04), with a large effect size (*d *= .96) suggesting high practical significance of children's perceived anxiety levels. Self-reported caregiver anxiety was also significantly less in the intervention group (STAI-S, p = 0.02) with a moderate effect size (*d *= .59).

Baseline heart rate and systolic blood pressure recorded at the outpatient clinic visit prior the manometry procedure were available for 74.6% and 77.8% of subjects, respectively. Of those with whom baseline data was available, comparative analysis was performed with physiological measures of arousal obtained just prior to the manometry procedure. For those in the intervention group, systolic blood pressure decreased an average of 6.7 mmHg from the baseline reading to the preprocedural measurement, whereas the systolic blood pressure of the controls increased an average of 2.9 mmHg, which was significantly different and with a moderate effect size (p <0.05, d = .66). While heart rate interval changes demonstrated a similar trend, the difference was not significantly different (p = 0.43).

After procedure completion, caregivers of patients in the intervention group reported significantly less observed distress compared with caregivers of subjects in the control group (p = 0.01). The staff gastroenterologist also reported significantly less observed distress of subjects in the intervention group (PBCL, p<0.01). Further, Cohen's effect size value (*d* = .66 and* d* = 1.12, respectively) suggested a moderate to high practical significance for these measured variables. Both variables were analyzed with a two-way ANOVA to determine if the child's age interacted with the main intervention effect. In both cases, there was no statistically significant difference in either PBCL scores (p = 0.33) or parent-reported observed distress (p = 0.49) between younger (≤7 years) or older (≥8 years) children. Relatively high procedural satisfaction was reported by caregivers in both groups, with no significant differences (p = 0.12). Results are detailed on Table 2.

## 4. Discussion

It is likely that healthcare providers underestimate the anxiety associated with anorectal manometry as it typically does not involve sedation or require IV placement. In a prior study of thirty-five children (mean age = 7.2 years, +/-2.8), we found that HRM results in significantly elevated anxiety and observed procedural distress [9]. These findings also identified elevated anxiety in the accompanying parent, indicating that anxiety is not limited to the patient. For both the child and parent, anxiety and procedural distress were comparable to more invasive procedures. For parents, this was similar to previously published data on adults undergoing endoscopic procedures themselves.

History of a distressing medical procedure during childhood has been linked to later somatization [1], increased self-reported procedural fear and pain as adults, and subsequent avoidance of medical situations [2, 3]. Therefore, increased efforts are being made to diminish procedural distress in children. There is a strong body of research supporting the effectiveness of psychoeducational preparation programs and behavioral distraction interventions with many types of medical procedures [16–19], including pediatric colonoscopy [4] and esophagogastroduodenoscopy [5]. Psychoeducation, or providing specific information about what will happen during a medical procedure and how it will look, feel, etc., can help reduce anticipatory anxiety, minimize distress during the procedure, optimize treatment outcomes, and result in better adjustment following the procedure [5, 16–18]. Blount et al. [20] recommend the preparatory information be specific rather than general and should include procedural as well as sensory information in order to accurately prepare the child.

Despite the extensive amount of research evaluating behavioral and psychological interventions of a variety of medical procedures, there have been no known studies published on the intervention of procedural anxiety during HRM. The anorectal manometry procedure is relatively unique in this way in that results can be significantly affected by the child's cooperation and level of distress. While the procedure can be performed while under general anesthesia to determine the presence of the RAIR, determination of rectal sensory volumes and functional measures are ideally performed while alert and in a cooperative state. The functional and sensory data gathered from HRM in the alert state can greatly influence medical, psychological, and surgical intervention of anorectal disorders. Therefore, it is important to develop and implement effective strategies to reduce a child's procedural distress during HRM.

The current study aimed to determine if a psychological preparation intervention (psychoeducation video) would decrease perceived and measured procedural distress in pediatric patients undergoing HRM. Results indicated children receiving the psychological intervention reported significantly less anxiety and demonstrated significantly less observed distress by both parent and physician rating. In addition, autonomic nervous system stimulation was also significantly decreased for the intervention group, as measured by changes in systolic blood pressure between baseline and immediately prior to HRM procedure. Results did not indicate a significant impact of the intervention on parent's satisfaction of the procedure itself, which is likely due to high ratings of satisfaction across both groups. High parental satisfaction despite significant child distress may reflect the common belief that medical procedures are expected to be distressing for children and thus these two variables may not be specifically related.

For all significant effects, the calculated effect sizes were generally medium to large, suggesting a moderate to high practical significance for each of these measured variables. The results are consistent with previous research identifying psychological preparation as an effective intervention in other gastroenterological medical procedures [4, 5] and expand on this research to include procedures that require active patient participation.

In addition to preparing the child, training the parent/caregiver in effective coping skills to enhance their use of coping-promoting behaviors is an effective intervention strategy [21, 22]. Several studies have shown that some parent and medical staff behaviors have been shown to be helpful to children (i.e., coping-promoting: nonprocedural talk, humor, and coaching), whereas other behaviors have been found to be detrimental (i.e., distress promoting: reassurance, empathy, apologies, giving control, and criticism) [20]. Distress promoting comments focus the child's attention on their own distress, thereby heightening his or her distress, whereas coping-promoting comments redirect his or her attention elsewhere [21].

The psychoeducation video was also found to significantly reduce parental anxiety, such that parents in the intervention group reported significantly less anxiety compared with the control group. As detailed in the methods section, the final 40% of the video focused on psychological preparation for caregivers, delineating their role during the procedure. Unlike endoscopic procedures, parents are typically permitted to remain with their child during the performance of unsedated HRM and even relied upon to assist in comforting to facilitate completion. It is likely that parental and child distress exacerbate each other, resulting in a cyclical pattern. The video specifically targeted parental anxiety and behaviors to indirectly affect the child's distress and procedural outcomes.

The specific intervention was minimal contact, in that the psychoeducation was provided via a short video, requiring no additional personal and minimal additional time to administer. The video itself was specifically designed to engage both pediatric audiences as well as their caregivers. Specifically, the video utilized peer modeling, bright colors, simplistic language, and cartoon features to detail the entire procedure from check-in until the child was leaving the clinic.

All subjects underwent procedures completed by the same one staff gastroenterologist and a single nurse, minimizing variability of the patient and caregiver experience. However, due to logistical constraints, while the study was randomized, the staff gastroenterologist was not blinded to group assignment, possibly affecting the PBCL results. Subjects were also randomized and those in the intervention group were shown the psychoeducation video immediately prior to the procedure, allowing for these children to only view the video one time after arriving in the clinic and potentially already facing elevated anxiety. Future interventions may be more beneficial if offered earlier in the process (e.g., prior to arriving to the clinic).

In conclusion, results of this randomized experiment expand on previous research finding benefits of psychological intervention in reducing distress during medical procedures. In the anorectal manometry procedure, patient distress and parent anxiety were both reduced with psychoeducational interventions and the procedure itself was more efficient and effective. Understanding and utilizing psychological methods that decrease distress for patients and their caregivers is imperative to improving medical care standards.

## Figures and Tables

**Table 1:** Demographics and baseline characteristics.

Variable	Control (N = 32)	Intervention (N = 31)	Overall (N = 63)	T Score / Chi-Square	P-value
Age (years)					

Mean (Standard Deviation)	6.68 (2.69)	6.69 (2.33)	6.68 (2.49)	-0.02	0.99

Minimum	3	3	3		

Maximum	12	12	12		

Gender				0.76	0.38

Male	20 (62.5%)	16 (51.6%)	36 (57.1%)		

Female	12 (37.5%)	15 (48.4%)	27 (42.9%)		

Race				0.69	0.71

White	22 (68.8%)	24 (77.4%)	46 (73.0%)		

Black	8 (25.0%)	6 (19.4%)	14 (22.2%)		

Other	2 (6.3%)	1 (3.2%)	3 (4.8%)		

Caregiver Accompanied With				0.24	0.89

Mother	32 (100%)	28 (90.3%)	60 (95.2%)		

Father	10 (31.3%)	11 (35.5%)	21 (33.3%)		

Multiple Caregivers	14 (43.8%)	12 (38.7%)	26 (41.2%)		

Psychiatric Comorbidity	7 (21.9%)	2 (6.5%)	9 (14.3%)	3.05	0.08

**Table 2:** Comparison of intervention to control group.

	Control Group		Intervention Group		T Score / Chi-Square	P-value	Effect Size
							(Cohen's D)
	*N*	Mean (SD)	*N*	Mean (SD)			
		N (%)		N (%)			

PBCL (total)	32	13.16 (6.36)	31	7.55 (3.19)	4.45	<0.01	1.12

STAIC-S	11	46.27 (3.85)	10	42.70 (3.56)	2.21	0.04	0.96

STAI-S	32	39.81 (13.26)	31	33.16 (8.79)	2.35	0.02	0.59

Parental Rating of Child Distress	32	2.19 (1.66)	30	1.20 (1.32)	2.61	0.01	0.66

Parental Satisfaction Rating	32	5.69 (.78)	30	5.60 (.68)	0.49	0.64	

HR Difference between baseline and immediately prior to beginning manometry	25	1.04 (15.21)	22	-2.23 (12.69)	0.80	0.43	

SBP Difference between baseline and immediately prior to beginning manometry	26	2.85 (11.29)	23	-6.65 (16.91)	2.28	0.03	0.66

Notes: PBCL = Procedure Behavior Checklist, STAI-C = State-Trait Anxiety Scale for Children-State, STAI-S = State-Trait Anxiety Scale-State, HR = heart rate, and SBP = systolic blood pressure.

## Data Availability

The data used to support the findings of this study are available from the corresponding author upon request.
